# Monocyte count is associated with the severity of human adenovirus pneumonia in hospitalized children aged less than 6 years

**DOI:** 10.1186/s12879-023-08036-y

**Published:** 2023-02-02

**Authors:** Rong Hu, Xiaorong Luo, Guilan Tang, Yiyi Ding

**Affiliations:** 1 Department of Pediatrics, The First People’s Hospital of Changde City, #818 Renmin Middle Road, Changde, 415003 China; 2 Department Clinical Laboratory, The First People’s Hospital of Changde City, Changde, 415003 China

**Keywords:** Children pneumonia, Human adenovirus, HAdV pneumonia, Monocytes, Monocyte count

## Abstract

**Background:**

Human Adenovirus (HAdV) pneumonia is common in young children and infants. Overall, 7–8% of all viral respiratory illnesses among children for less than 5 years are induced by HAdVs. Unfortunately little is known about the role of monocyte count in the disease severity.

**Methods:**

Data were gathered from 595 children (age < 6 years) who were diagnosed with HAdV infection at the 1st People's Hospital (Changde City, China) between January 2019 and December 2019. There were 181 cases of severe adenovirus pneumonia.

**Results:**

The correlation between the patients' monocyte count and the severity of HAdV pneumonia was estimated by performing a multiple linear regression analysis. The results showed a negative association (OR: 0.53, 95% CI 0.31 to 0.89, P < 0.05). We further built Generalized Additive Models (GAMs) and demonstrated that the monocyte count had a non-linear association with severe HAdV pneumonia. The inflection point of monocyte count detected in the two-stage linear regression model was 1.5. On the left side of this point, the monocyte count was negatively interrelated (OR: 0.26, 95% CI 0.13 to 0.52, P < 0.001), while on the opposite side, there was a positive association (OR: 7.48, 95% CI 1.30 to 43.08, P < 0.05).

**Conclusions:**

Based on the results of this investigation, we established a link between monocyte count and the severity of HAdV pneumonia. Monocyte count is negatively associated with severe HAdV pneumonia. The inflection point of monocyte count detected in the two-stage linear regression model was 1.5 × 10^9^/L.

## Background

Human adenoviruses (HAdVs) can often cause infection in young children. The HAdVs lack the envelope and they possess double-stranded DNA. In young children and infants, HAdVs initiate complications that resemble cold, rhinitis, fever, cough, and aching throat. Unfortunately, when these infections spread to the lungs, they induce bronchitis, bronchiolitis, and pneumonia. These infections most commonly affect children. Overall, 7–8% of all viral respiratory illnesses among children for less than 5 years are induced by HAdVs, and the outcome can be austere, even lethal [1, 2]. HAdVs-induced respiratory infections account for 2–5% of all respiratory tract infections and 4–10% of all types of pneumonia [3]. These statistics make the necessity of diagnosing, evaluating, and predicting the severity of these infections to develop and provide solid and on-time health care for managing infant HAdV pneumonia.

Data indicate a HAdV pandemic among young children in Southeastern China from the winter of 2018 to the end of 2019 [4]. Based on the experience of our pediatric clinicians, the incidence of HAdV infection among outpatient and hospitalized children in Changde aslo reached an outbreak in 2019. During this period, many children with respiratory infections were reported at the Pediatric Outpatient Clinic at the 1st People's Hospital (Changde City, China). Patients who tested positive for HAdV antibodies in the outpatient clinic were also detected. We found that along with the routine blood tests, some patients had a significantly increased monocyte count compared with the reference values. As a result, we decided to investigate whether the higher monocyte count was associated with a mild disease history that would suggest avoiding subsequent hospital admission.

Monocytes are part of the macrophage family in the blood. Their functions are not relatively well-understood. It is known that monocytes are involved in inflammation, injury and infection as they are connected with endothelia and can penetrate tissues at the sites of infection, thus affecting endothelial cell permeability and angiogenesis [5]. Some studies report that monocytes lessen inflammation by discharging immune suppressive cytokines and enzyme products that release pathogens. These findings indicate a vital function for this type of blood cell in the innate immune response against microbial infection. They also imply that they play a role in enduring immunity and adaptive immune responses [6, 7]. The findings of a study on HAdV-induced pneumonia in healthy adult patients showed that patients with respiratory failure (RF) had significantly lower (P < 0.001) absolute and percentage counts of monocytes than patients without RF. The authors assumed that the detected initial monocytopenia was significant for predicting RF [8]. Some data highlight the association of monocyte count with Klebsiella pneumonia, dengue fever, human respiratory syncytial virus and other chronic infections [7, 9–11]. The processes by which this correlation works, however, remain obscure. Furthermore, it is unclear how this monocyte count relates to the severity of HAdV-induced pneumonia in children.

This study aims to investigate the relationship between monocyte count and the severity of HAdV pneumonia and to develop it as a predictive factor for adenovirus infections, particularly in children under the age of six who have severe HAdV pneumonia.

## Methods

### Subjects

Patients' data were collected from the electronic medical records of the 1st People's Hospital (Changde City, China) and were processed anonymously to guarantee privacy. The study was approved (Approval No. 2020-145-01) by the Ethics Committee of the 1st People's Hospital (Changde City, China) and followed the guidelines of Ethics (Declaration of Helsinki). 595 pediatric patients were examined by a medical specialist between January 2019 to December 2019 were diagnosed with HAdV pneumonia following strict pneumonia diagnosis guidelines available by the World Health Organization (WHO) [12]. The diagnosis was further confirmed by positive multiplex polymerase chain reaction (PCR) for HAdVs in the nasopharynx of all patients admitted within 24 h. Clinical data were obtained by screening medical records, including symptom manifestations, standard tests, and diagnostic data.

All 595 participants were assigned to two groups: (1) a group with mild HAdV infection (including patients with an upper respiratory infection, bronchiolitis and mild pneumonia); (2) a group with severe HAdV pneumonia. The pneumonia severity classification was based on the WHO guidelines [12]. In severe pneumonia, oxygen was administered, and the diagnosis was performed when cough or difficulty breathing were present, with ≥ 1 of the listed symptoms like lower chest wall draws in with each breath, flaring of nostrils, or grunting (in young infants).

### Exclusion and inclusion criteria

Inclusion criteria: (1) patients between 28 days and 6 years old. (2) Admitted to the pediatric department of the 1st People's Hospital (Changde City, China) from January 2019 to December 2019. (3) With positive PCR detection of HAdV in patient nasopharyngeal swab samples.

Exclusion criteria: (1) patients with HIV infection, malignant disease, diagnosed or suspected tuberculosis, ongoing immunosuppression therapy, immunodeficiency, severe organ dysfunction, chronic illness like a congenital heart or chronic lung disease. (2) patients with substantial missing medical data, and (3) with abnormally high monocyte count (two cases were excluded from the study. One case was a 4-month-old infant diagnosed with severe HAdV pneumonia with a monocyte count of 4.9 × 10^9^/L; the other was a 1-year-old child diagnosed with bronchiolitis with a monocyte count of 3.4 × 10^9^/L).

### Data evaluation and statistical examination

Continuous variables were divided into two groups: normally and non-normally distributed. The normally distributed are presented as means ± standard deviations, while the non-normally as medians with interquartile ranges (Q1, Q3). On the other hand, categorical variables are expressed percentages or occurrences. The χ^2^ test was used to compare rates. The t-test (for data with a normal distribution) and nonparametric Wilcoxon rank-sum test were applied to intergroup comparisons (data with abnormal distribution). Variables that showed an independent association with severe HAdV pneumonia in the univariate analysis were analyzed further by multifactor analyses. The PCR data distribution was lopsided to the left. Therefore, we performed a Log10 transformation (LgHAdV) on these data before the examination. Univariate and Logistic regression models were further conducted to calculate the odds ratios (OR) and 95% confidence intervals (CI). They allowed evaluation of the possible relations between monocyte count and the risk of developing severe HAdV pneumonia. Multifactor Logistic regression analysis uses three models. The first two models were Model 1 (non-adjusted) and 2 (adjusted for sex and age). Model 3 combined Model 2 with other covariates as show in Table 3. We used the same multifactor logistic regression model analysis to test the results' stability. The monocyte counts were used as continuous, four categorical, and trend variables to verify the result.

Additionally, the general combinatorial model was used to distinguish between the non-linear relationship between monocyte count and the risk of developing severe HAdV pneumonia. Smooth curve fitting and a weighted general additive model were used to demonstrate the potential nonlinearity of the data. Moreover, two-piecewise linear regression models demonstrated thresholds in non-linear associations. The EmpowerStats software (www.empowerstats.com, X&Y solutions, Inc, Boston, MA) was used for statistical analyses and the two-sided P values of less than 0.05 were designated as statistically significant.

## Results

### Participants' medical data and characteristics

A total of 595 patients were included in the present study. They were assigned to two groups: patients diagnosed with upper respiratory infections, bronchiolitis, and mild pneumonia, one group (mild HAdV infection group), and the other severe HAdV pneumonia group. We have performed statistical analysis with these data. The estimated mean age was 2.4 ± 1.5 years. 61.2% were males, and 38.8% were females. 17.6% of these children were less than 1 year old. 181 patients were finally diagnosed with severe HAdV pneumonia, of whom 10 (5.5%) required mechanical ventilation, 69 (38.1%) needed continuous positive airway pressure mode, and 102 (56.4%) were supplemented with oxygen. When examining patients' age, weight, length of hospital stay, red blood cell count, monocyte count, neutrophil count, the blood serum levels of aspartate aminotransferase (AST), creatine kinase isoenzyme (CKMB), procalcitonin (PCT), and other blood serum parameters, we found statistically significant differences (P < 0.05) among the patients (Table 1). We did not detect significant differences among the patients from the two groups in terms of sex, weight at birth, eutocia, fever, white blood cell, platelet, and LgHAdV (P > 0.05) (Table 1).

**Table 1:** HAdV infectious patient characteristics

Variable	Mild HAdV infection (n = 414)	Sever HAdV Pneumonia (n = 181)	P-value
Demographic data			
Age, Median(IQR)[years]	3.0 (1.0–4.0)	1.0 (0.8–2.0)	< 0.001
Weight, Mean ± SD [kg]	14.3 ± 4.0	11.3 ± 3.5	< 0.001
Bornweight, Mean ± SD [g]	3271 ± 506	3269 ± 566	0.966
Length in hospital, Mean ± SD [days]	6.2 ± 2.8	12.0 ± 5.6	< 0.001
Male, n (%)	246 (59.4%)	118 (65.2%)	0.184
Eutocia, n (%)	191 (46.5%)	80 (44.7%)	0.690
Fever, n (%)	409 (98.8%)	175(96.7%)	0.079
Laboratory data			
White blood cell, Mean ± SD [× 10^9/L]	8.5 ± 4.0	9.1 ± 5.3	0.155
Red blood cell, Mean ± SD [× 10^12/L]	4.3 ± 0.4	4.2 ± 0.5	0.003
Hemoglobin, Mean ± SD [g/L]	113.7 ± 10.9	108.5 ± 11.3	< 0.001
Platelet, Mean ± SD [10^9/L]	254.1 ± 100.0	270.8 ± 141.4	0.100
Lymphocyte, Median(IQR) [× 10^9/L]	2.8 (2.0–3.8)	2.9 (1.8–4.2)	0.726
Neutrophils, Median(IQR) [× 10^9/L]	3.3 (1.9–5.9)	4.0 (2.6–6.4)	0.013
Monocyte, Median(IQR) [× 10^9/L]	0.7 (0.5–1.1)	0.5 (0.3–0.9)	< 0.001
Albumin, Mean ± SD [g/L]	39.6 ± 2.9	37.7 ± 4.3	< 0.001
ALT, Median(IQR) [U/L]	14.0 (11.0–19.0)	20.0 (14.0–28.0)	< 0.001
AST, Median(IQR) [U/L]	38.0 (32.0–49.0)	58.0 (44.0–88.0)	< 0.001
CKMB, Median(IQR) [U/L]	24.6 (19.9–31.1)	31.0 (25.4–44.5)	< 0.001
LgHAdV, Mean ± SD	5.9 ± 1.6	5.8 ± 1.9	0.378
CRP, Median(IQR) [mg/L]	12.8 (4.2–28.2)	11.6 (4.2–26.2)	0.671
PCT, Median(IQR) [ng/ml]	0.2 (0.1–0.5)	0.4 (0.1–0.8)	< 0.001
Therapeutic measures			
Methylprednisolone used, n (%)	60 (14.5%)	130 (71.8%)	< 0.001
Gammaglobulin, n (%)	39 (9.4%)	125 (69.1%)	< 0.001
Fibrobronchoscopy, n (%)	33 (8.0%)	112 (61.9%)	< 0.001
Respiratory support, n (%)			< 0.001
Unused	414 (100.0%)	0 (0.0%)	
Oxygen	0 (0.0%)	102 (56.4%)	
CPAP	0 (0.0%)	69 (38.1%)	
MV	0 (0.0%)	10 (5.5%)	

IQR: interquartile range, Q1–Q3; ALT: alanine aminotransferase; AST: aspartate aminotransferase; CKMB: creatine kinase isoenzyme; LgHAdV: Log10 transformation for Human Adenovirus of Real-Time PCR Quantitative; CRP: C-reactive protein; PCT: procalcitonin

### Univariate statistical analysis

We have performed univariate analyses with patients' data and the results are displayed in Table 2. The binary logistic regression analysis results showed that variables such as the patient's sex, the level of C-reactive protein (CRP) in their blood serum, and the presence of LgHAdV did not show an association with the diagnosis of severe HAdV pneumonia. The results also showed that the blood serum concentrations of AST, CKMB, and methylprednisolone used were positively associated with severe HAdV pneumonia. In contrast, parameters like age, red blood cells count, and monocyte count were negatively associated with severe HAdV pneumonia (P < 0.05, Table 2).

**Table 2:** Univariate analyses for severe HAdV pneumonia

Variable	Mean ± SD/Median (IQR)/n (%)	OR	95% CI	*P*-value
Female	231 (38.8%)	0.78	(0.54, 1.12)	0.184
Age, [years]	2.0 (1.0–4.0)	0.57	(0.50, 0.66)	< 0.001
Red blood cell, [× 10^12/L]	4.3 ± 0.4	0.53	(0.35, 0.81)	0.003
Monocyte,[× 10^9/L]	0.7 (0.4–1.0)	0.52	(0.35, 0.77)	0.001
ALB, [g/L]	39.0 ± 3.5	0.85	(0.81, 0.90)	< 0.001
ALT, [U/L]	15.0 (12.0–23.0)	1.01	(1.00, 1.02)	0.002
AST, [U/L]	42.0 (33.0–57.0)	1.03	(1.02, 1.04)	< 0.001
CKMB, [U/L]	26.5 (20.9–34.1)	1.05	(1.04, 1.06)	< 0.001
CRP, [mg/L]	12.2 (4.2–27.6)	1.00	(0.99, 1.01)	0.670
PCT, [ng/ml]	0.2 (0.1–0.7)	1.35	(1.11, 1.65)	0.003
LgHAdV	5.9 ± 1.7	0.96	(0.86, 1.06)	0.377

IQR: interquartile range, Q1–Q3

Result variable: severe HAdV pneumonia

### Data of adjusted and unadjusted binary logistic regression of patients' parameters and characteristics

We have investigated the association between the monocyte count and the severity of HAdV pneumonia, and we have created both not-adjusted and adjusted models (Table 3). The monocyte count presented an inverse association with severe HAdV pneumonia (OR: 0.52, 95% CI 0.35 to 0.77, P < 0.01) in the basic model 1. We have compared Model 2, in which we applied statistical adjustment for parameters like sex and age, with the basic one and found no big differences (OR: 0.36, 95% CI 0.24 to 0.55, P < 0.001). Model 3, in which the adjustment included all studied variables, showed a similar correlation (OR: 0.53, 95% CI 0.31, 0.89, P < 0.05). This means that the probability of developing severe HAdV pneumonia was lowered by 47% for every increase in monocyte count in a patient with 10^9^/L. We used the monocyte count as a categorical variable (quartile) to further validate these findings. In which we compared these data with the Q1 (reference group), the estimated OR value for the risk of severe HAdV pneumonia in the Q2, Q3, and Q4 did not appear stable, respectively. The results showed that the trend between the quartile of monocyte count was significant (P < 0.001). With the results of the monocyte count as a continuous variable, these results did not seem to be credible. The non-equidistant range of the effect magnitude in Table 3 reveals that the relationship between the monocyte count and the severity of HAdV pneumonia was not linear.

**Table 3:** Multifactor Logistic regression analysis for severe HAdV pneumonia

Exposure	Model 1	Mode 2	Model 3
Monocyte	0.52 (0.35, 0.77) 0.001	0.36 (0.24, 0.55) < 0.001	0.53 (0.31, 0.89) 0.016
Monocyte(quartile)			
Q1	Ref	Ref	Ref
Q2	0.37 (0.23, 0.61) < 0.001	0.32 (0.19, 0.56) < 0.001	0.41 (0.21, 0.81) 0.010
Q3	0.26 (0.16, 0.44) < 0.001	0.19 (0.11, 0.33) < 0.001	0.22 (0.11, 0.45) < 0.001
Q4	0.36 (0.22, 0.59) < 0.001	0.22 (0.13, 0.38) < 0.001	0.36 (0.18, 0.71) 0.003
P for pretend	< 0.001	< 0.001	0.001

Data in the table: OR (95% CI) P value

Result variable: severe HAdV pneumonia; Exposure variables: Monocyte

Model 1: adjust for none

Mode 2: adjust for sex, age

Model 3: adjust for sex, age, Red blood cell, CKMB, AST, LgHAdV, fever, and methylprednisolone used

### Non-linear relationship between monocyte count and severe HAdV pneumonia

**Fig. 1:**
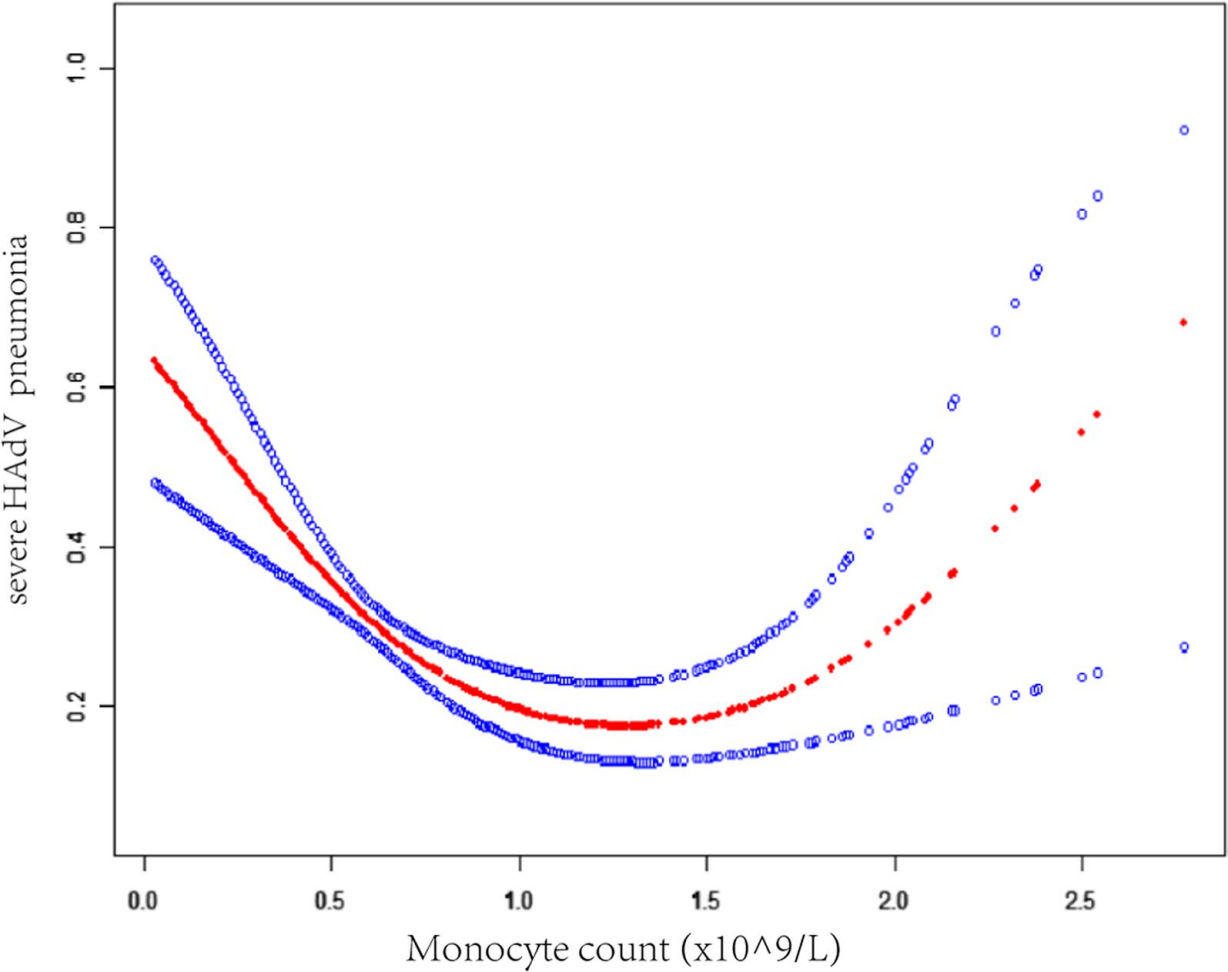
Association between the monocyte count and severe HAdV pneumonia. A threshold, non-linear association between the monocyte count and severe HAdV pneumonia was detected (P < 0.001) in a generalized additive model (GAM). The solid red line represents the smooth curve fit between variables. Blue bands represent the 95% of confidence interval from the fit. All were adjusted for sex, age, Red blood cell, CKMB, AST, LgHAdV, fever, and methylprednisolone used

Figure 1 demonstrates the non-linear relationship between the monocyte count and the severity of HAdV pneumonia. We have assumed the monocyte count as an unbroken adjustable, and we have further built a generalized additive model (GAM) to review whether there was a non-linear relationship between the monocyte count and the severity of the studied type of pneumonia. The built smooth curve adjusted for parameters like sex, age, red blood cell, CKMB, AST, LgHAdV, fever and methylprednisolone used through GAM proved that the monocyte count had a non-linear relationship with severe HAdV pneumonia (Fig. 1). The linear regression and two-stage linear regression models were compared. We proved that the detected association between the monocyte count and severe HAdV pneumonia was significant (P = 0.003) (Table 4). The detected inflection point of the monocyte count was 1.5 (× 10^9/L), as shown by the two-stage linear regression exemplary. We further observed that on the left side of this point (n = 535), the monocyte count and the severity of the studied pneumonia were negatively connected (OR: 0.26, 95% CI 0.13 to 0.52, P < 0.001), while on the opposite side (n = 60), they were positively associated (OR: 7.48, 95% CI 1.30 to 43.08, P < 0.05) (Fig. 1, Table 4).

**Table 4:** Nonlinearity addressing of monocyte and severity of adenovirus infection

Outcome:	OR (95% CI)	*P*-value
Model 1 Fitting model by standard linear regression	0.526 (0.312, 0.888)	0.016
Model 2 Fitting model by two-piecewise linear regression		
Inflection point	1.5	
< 1.5	0.257 (0.126, 0.524)	< 0.001
> 1.5	7.478 (1.298, 43.079)	0.024
P for the log-likelihood ratio test		0.003

Adjustment variables: sex; age; Red blood cell; CKMB; AST; LgHAdV; fever; methylprednisolone used

## Discussion

In the present investigation, respiratory HAdV infection in children younger than 6 years old was characterized. We evaluated the potential relationship between the monocyte count of the patients and the disease severity. There is a shortage of information about this association in the literature, particularly for young children. Implying that the monocyte count was connected to the seriousness of HAdV infection possible after the 1st People's Hospital in Changde City, China, observed various degrees of increased monocyte counts in outpatients and inpatients with HAdV infection. Our data showed such a correlation via the performed multiple regression data. Interestingly, the smooth curve fit (Fig. 1) and the two-piece-wise linear regression model (Table 4) showed that the count of monocyte less than the detected infection point of < 1.5 × 10^9^/L were negatively associated with severe HAdV pneumonia.

Some authors claimed that primary monocytopenia could potentiate the prognosis of future respiratory failure during HAdV pneumonia in patients without compromising their immunity [8]. Another study included data from 80 community-acquired pneumonia patients and proved that the monocyte count in the liver injury group was lesser than that of the normal one (P < 0.05) [13]. Similarly, other authors established that the reduction in the monocyte count was linked with a tenfold escalation in the bacterial colony-forming units (CFUs) in the pulmonary tract. In contrast, neutrophil exhaustion was associated with little reduction in these CFUs. Additional evidence showed that bacterial propagation spread into the mediastinal lymph nodes and spleen in monocyte-depleted mice was also accelerated [9]. The effectiveness and speed of bacterial eradication were also hampered. Data point out that the monocytes deliver protection against infections with *Klebsiella pneumoniae* [9].

Furthermore, other authors proved that there was a negative correlation between the monocyte count and the 21-day probability of lung failure in patients that were prior diagnosed with HAdV pneumonia and treated with Cidofovir [14]. Lower levels of monocytes appear to be associated with a more unfavourable disease condition, which is consistent with our results. Although monocytes provide possible immune protection mainly against different infections or severe HAdV pneumonia, excessive monocyte infiltration has been reported to compromise immunity in mice with uncontrolled influenza A virus reproduction, demonstrating an extreme monocyte infiltration into the lungs [15]. Our results which showed that monocyte count higher than 1.5 × 10^9^/L were positively associated with severe HAdV pneumonia (Fig. 1, Table 4), appear to support this view.

Monocytes originate in the bone marrow from common ancestor cells shared with granulocytes and macrophages. In addition, the recruitment of these cells is vital for the effectively managing and eliminating viral infections [11]. These cells are the initial types of immune system cells interacting with pathogens. On the other hand, these cells may also be infected and act as virus shuttles, facilitating the spread of viruses [16, 17]. Blood circulating monocytes are predecessors for tissue-specific macrophages and other types of dendritic cells both in vitro and in vivo. The microbial infection triggers in vivo monocyte specialization into specific dendritic cells that improve the management of any disease [6]. There is strong evidence that mononuclear phagocytes restrict viral release, recognize and phagocytose pathogens, clear viral and apoptotic cells, start generating cytokines to modulate inflammation, and have a protective function during influenza A virus and respiratory syncytial virus infections [11, 18]. One study reported that the degree of T-cell suppression and cytotoxicity in children with non-severe HAdV pneumonia was less compared to children with severe adenoviral pneumonia. The CD3+, CD4+, and CD4+/CD8+ ratio values were higher than the severe HAdV pneumonia group. The levels of IL-2, IL-6, IL-10, and tumor necrosis factor-α of children in the severe group were higher than those in the non-severe group. The immunophenotype of peripheral blood T lymphocytes and cytokines could help evaluate the severity of HAdV pneumonia [19].

Similarly, another report also suggested that infection biomarkers, such as procalcitonin, were significantly increased, and the absolute counts of CD3+, CD4+, CD8+ T cells, and NK cells were significantly reduced in severe HAdV infection patients compared with mild patients [20]. Macrophages are essential cells of the innate immune, and they can adapt under both pro- and anti-inflammatory conditions and develop different functions. A growing body of evidence regarding a novel macrophage subpopulation that expresses CD3 has recently emerged. The human circulating monocytes can be differentiated into CD3+ TCRαβ+ and CD3+ TCRαβ− macrophages [21]. This might be one factor why higher monocytes are associated with milder HAdV infection.

Our research has some limitations. Because these co-infections are uncommon in our clinical practice during the local epidemic season, we haven't collected information on co-infections associated with monocytes. The presented statistical analyses include small samples (n = 60) when monocytes > 1.5 × 10^9^/L, and the results might be biased. Without addressing the relationship between monocyte count and the disease in the upper or lower respiratory tract, our primary goal was to evaluate the relationship between monocyte count and the severity of HAdV pneumonia. Further studies are needed to prove whether monocyte count is associated with worse disease outcomes in patients with outpatient primary HAdV respiratory infections and to predict whether HAdV outpatients with fever will require further medical intervention or only be observed at home.

## Conclusions

Our results prove the negative association between monocyte count and severe HAdV pneumonia in children under 6 years at monocyte counts less than 1.5 × 10^9^/L. Monocyte count may be a protective factor for severe HAdV infection in infants and young children. Furthermore, it could supplement the initial screening of children with early adenovirus infection when examined in an outpatient clinic to avoid overcrowding medical resources. As a result, relevant determinants of severe adenovirus infection should be explored prospectively.

## Data Availability

The datasets used and analyzed during the current study are available from the corresponding author upon reasonable request.

## Abbreviations

HAdV: Human adenovirus
GAMs: Generalized Additive Models
RF: Respiratory failure
WHO: The World Health Organization
PCR: Multiplex polymerase chain reaction
LgHAdV: Log10 transformation for Human Adenovirus of Real-Time PCR Quantitative
OR: Odds ratios
CI: Confidence intervals
AST: Aspartate aminotransferase
CKMB: Creatine kinase isoenzyme
PCT: Procalcitonin
CRP: C-reactive protein
CFUs: The bacterial colony-forming units
